# Association Between Endometriosis Phenotype and Preterm Birth in France

**DOI:** 10.1001/jamanetworkopen.2021.47788

**Published:** 2022-02-08

**Authors:** Louis Marcellin, Francois Goffinet, Elie Azria, Anne Thomin, Charles Garabedian, Jeanne Sibiude, Eric Verspyck, Martin Koskas, Pietro Santulli, Jessica Rousseau, Pierre-Yves Ancel, Charles Chapron

**Affiliations:** 1 Université de Paris, Paris, France; 2 Département de Gynécologie Obstétrique II et Médecine de la Reproduction, Assistance Publique-Hôpitaux de Paris (AP-HP), Centre Hospitalier Universitaire (CHU) Cochin, Hôpital Universitaire Paris Centre (HUPC), Paris, France; 3 Port-Royal Maternity, AP-HP, Centre-University of Paris, Federation Hospitalo-Universitaire for Prematurity, Paris, France; 4 Maternité Notre-Dame-de-Bon-Secours, Groupe Hospitalier Paris-Saint-Joseph, Paris, France; 5 Sorbonne Université, Paris, France; 6 Trousseau Hospital, AP-HP, Paris, France; 7 University Lille, Unité Lilloise de Recherche 2694, Évaluation des Technologies de Santé et des Pratiques Médicales, Lille, France; 8 CHU Lille, Department of Obstetrics, Lille, France; 9 Service de Gynécologie-Obstétrique, AP-HP Hôpital Louis Mourier, Colombes, France; 10 Service de Gynécologie et Obstétrique, CHU de Rouen, Rouen, France; 11 Department of Gynecology, Hôpital Bichat, AP-HP, Paris, France; 12 Clinical Research Unit, Center for Clinical Investigation P1419, AP-HP, Paris, France; 13 Centre de Recherche Épidémiologiques et Bio Statistiques de Sorbonne Paris Cité, Obstetrical Perinatal and Pediatric Epidemiology Research Team, Institut National de la Santé et de la Recherche Médicale, Institut National de la Recherche Agronomique, Paris, France

## Abstract

**Importance:**

Endometriosis is an inflammatory disease with a heterogeneous presentation that affects women of childbearing age. Given the limitations of previous retrospective studies, it is still unclear whether endometriosis has adverse implications for pregnancy outcomes.

**Objective:**

To evaluate the association between the presence of endometriosis and preterm birth and whether the risk varied according to the disease phenotype.

**Design, Setting, and Participants:**

This cohort study with exposed and unexposed groups was conducted in 7 maternity units in France from February 4, 2016, to June 28, 2018. Participants included women with singleton pregnancies who were followed up before 22 weeks’ gestation along with their newborns delivered at or after 22 weeks’ gestation. The final follow-up occurred in July 2019. Data were analyzed from October 7, 2020, to February 7, 2021.

**Exposures:**

Women in the endometriosis group had a documented history of endometriosis and were classified according to 3 endometriosis phenotypes: isolated superficial peritoneal endometriosis (SUP), ovarian endometrioma (OMA; potentially associated with SUP), and deep endometriosis (DE; potentially associated with SUP and OMA). Women in the control group did not have a history of clinical symptoms of endometriosis before their current pregnancy.

**Main Outcomes and Measures:**

The primary outcome was preterm birth between 22 weeks and 36 weeks 6 days of gestation. Association between endometriosis and the primary outcome was assessed through univariate and multivariate logistic regression analyses and was adjusted for the following risk factors associated with preterm birth: maternal age, body mass index (calculated as weight in kilograms divided by height in meters squared) before pregnancy, country of birth, parity, previous cesarean delivery, history of myomectomy and hysteroscopy, and preterm birth. The same analysis was performed according to the 3 endometriosis phenotypes (SUP, OMA, and DE).

**Results:**

Of the 1351 study participants (mean [SD] age, 32.9 [5.0] years) who had a singleton delivery after 22 weeks of gestation, 470 were assigned to the endometriosis group (48 had SUP [10.2%], 83 had OMA [17.7%], and 339 had DE [72.1%]) and 881 were assigned to the control group. No difference was observed in the rate of preterm deliveries before 37 weeks 0 days of gestation between the endometriosis and control groups (34 of 470 [7.2%] vs 53 of 881 [6.0%]; *P* = .38). After adjusting for confounding factors, endometriosis was not associated with preterm birth before 37 weeks’ gestation (adjusted odds ratio, 1.07; 95% CI, 0.64-1.77). The results were comparable for the different disease phenotypes (SUP: 6.2% [3 of 48]; OMA: 7.2% [6 of 83]; and DE: 7.4% [25 of 339]; *P* = .84).

**Conclusions and Relevance:**

This cohort study found no association between endometriosis and preterm birth, and the disease phenotype did not appear to alter the result. Monitoring the pregnancy beyond the normal protocols or changing management strategies for women with endometriosis may not be warranted to prevent preterm birth.

## Key Points

**Question:**

Is endometriosis associated with preterm birth?

**Findings:**

In this cohort study of 1351 women with or without endometriosis, endometriosis was not significantly associated with preterm birth after adjusting for the risk factors for preterm delivery. Disease phenotype did not appear to alter the result.

**Meaning:**

The findings suggest that modification of pregnancy monitoring or management strategies to prevent preterm birth for women with endometriosis may not be needed.

## Teaser

This cohort study examines the association of 3 endometriosis phenotypes with delivery before 37 weeks’ gestation among pregnant women with endometriosis.

## Introduction

Endometriosis is an inflammatory disease that affects 10% to 15% of women of reproductive age worldwide.^1^ Previous studies have yielded conflicting results regarding the implication of endometriosis for pregnancy outcomes.^2^ Endometriosis, which is defined as the presence of endometrial-like tissue outside the uterine cavity,^3^ has a heterogeneous clinical appearance consisting of 3 distinct phenotypes: isolated superficial peritoneal endometriosis (SUP), ovarian endometrioma (OMA) (potentially associated with SUP), and deep endometriosis (DE; potentially associated with SUP and OMA).^4,5^ Various hypotheses have been proposed to explain the adverse implications of endometriosis for pregnancy, but the pathogenesis and the specific role of each disease phenotype remain unclear. Endometriosis is believed to adversely affect uterine functions, especially by compromising properties of the eutopic endometrium.^6^ These alterations could interfere with the normal process of decidualization that helps control trophoblast invasion at the maternal-fetal interface,^7,8,9,10,11,12^ thereby potentially leading to severe placentation defects and obstetric complications.^10,13^ Considering endometriosis as an endometrial disease, a previous study documented the molecular alteration of the choriodecidual layer in women with DE, with molecular changes at the fetal-maternal interface.^14^

Three meta-analyses,^15,16,17^ including hospital retrospective studies, and 3 population-based retrospective cohort studies^18,19,20^ provide inconsistent results regarding the association between endometriosis and preterm birth, premature rupture of membranes, small for gestational age (SGA), preeclampsia, placenta previa, and cesarean delivery; however, the outcome of the disease phenotype has so far undergone only cursory assessment. These heterogeneous results may be attributed to several limitations of these studies, including their retrospective design, a lack of adjustment for confounders (assisted reproductive technology [ART], associated comorbidities, and multiple pregnancies), a lack of adequate control participants, and other methodological flaws.^21^ In the Endometriosis Obstetric (ENDOBST) study, we evaluated the association between the presence of endometriosis and preterm birth (before 37 weeks’ gestation) and whether the risk varied according to the disease phenotype.

## Methods

The ENDOBST study was a multicenter, prospective cohort study with exposed and unexposed groups. It was conducted in 7 academic maternity units in France (Maternité Port Royal, Hôpital Cochin, Paris; Maternité Notre-Dame-de-Bon-Secours, Hôpital Saint Joseph, Paris; Maternité Hôpital Louis Mourier, Colombe; Maternité Hôpital Bichat, Paris; Maternité Hôpital Trousseau, Paris; Maternité Charles-Nicolle, Rouen; and Maternité Jeanne de Flandre, Lille) from February 4, 2016, to June 28, 2018. All of the maternity units annually deliver newborns from more than 2000 women. Recruitment and data collection for this study commenced only after the female patients from these units had received information and provided signed informed consent to participate. The study was approved by the National Data Protection Authority and by the relevant Committee for the Protection of People Participating in Biomedical Research at each maternity unit. We followed the Strengthening the Reporting of Observational Studies in Epidemiology (STROBE) reporting guideline.

### Patient Selection

To be included in the study, patients had to be receiving health care for a single pregnancy, with follow-up before 22 weeks 0 days of gestation, and had to have delivered in any of the maternity units. The exclusion criteria were patients with a multiple pregnancy, an HIV-positive status, a referral from another hospital for maternal-fetal complications, and an opposition to the use of their medical data for research purposes. Neonates delivered at or after 22 weeks’ gestation were also included.

The exposed group (endometriosis group) comprised pregnant women with a history of endometriosis, which was documented retrospectively from an imaging workup using specific criteria to ascertain the diagnosis and the staging of the disease^22,23^ or from an available operative report describing endometriotic lesions with histological confirmation. At imaging, uterosacral endometriosis appeared as an irregular, hypoechoic thickening on ultrasonography and as an ill-defined infiltrative tissue that was hypointense on T2-weighted magnetic resonance imaging scans.^24^ At histological examination, the endometriosis was considered to be DE when the muscularis (regardless of the location: bladder, intestine, or intrinsic ureter) was infiltrated by endometriotic tissue after radical surgery (eg, bowel resection, partial cystectomy, or ureteral resection).^25^ For other locations (ie, uterosacral ligament, extrinsic ureter, or vagina), DE was arbitrarily defined as endometriotic tissue that was infiltrating beneath the surface of the peritoneum by greater than 5 mm.^26^ Because the 3 phenotypes of endometriotic lesions are frequently associated, patients were classified according to the worst lesions from least to most severe: isolated SUP, OMA (potentially associated with SUP), and DE (potentially associated with SUP and OMA).^27^ All reports were carefully examined to allow exact classification of the participants according to the disease phenotype.^22,28^

The control (unexposed) group comprised pregnant women who did not have clinical symptoms or a history of endometriosis. To avoid including women with undiagnosed endometriosis in the control group, we selected only those who did not have a history of school absenteeism for dysmenorrhea or a history of hormonal contraception prescription for intense primary dysmenorrhea, and those with no deep dyspareunia; such medical questions are relevant or specific to endometriosis.^1^

For each woman in the endometriosis group, we included 2 women in the control group from the same center. This approach enabled us to maintain the objective of a 1:2 exposed vs nonexposed ratio.

### Patient Follow-up

All patients had a first-trimester ultrasonography, and the gestational age was confirmed by ultrasonographic measurement of the craniocaudal length between 11 weeks and 13 weeks and 6 days of gestation. When the pregnancy was the result of ART, the gestational age was ascertained according to the date of the embryo transfer while taking the embryo stage into account. Prenatal ultrasonography was performed at least 3 times during each pregnancy, as recommended in France.^29^ Patients had a monthly checkup with a practitioner (obstetrician or midwife) and up to 36 weeks of amenorrhea. The final follow-up occurred in July 2019. The practices and the clinical decision-making regarding management of obstetric complications, labor, and delivery were comparable and consistent with the French recommendations for a normal pregnancy,^30^ preterm labor,^31^ hypertensive disorders,^32^ and intrauterine growth restriction.^33^

### Data Collection

Patients were enrolled prospectively before 22 weeks’ gestation during prenatal consultations. After obtaining signed written consent, the practitioners used the patients’ medical records to complete a detailed, paper-based questionnaire about maternal medical history, painful symptoms before the pregnancy, and the mode of conception. After delivery, clinical research assistants used the medical records to complete a detailed, web-based questionnaire regarding maternal characteristics, medical history, pregnancy course and complications, delivery, and postpartum events as well as neonatal health.

For each patient, we collected data that were potentially related to preterm birth, including general characteristics (age, body mass index [calculated as weight in kilograms divided by height in meters squared], tobacco use, country of birth, educational level, and professional activity during pregnancy), medical-surgical history (eg, preexisting diabetes and hypertension, history of gynecological surgery), obstetric history (eg, pregnancy loss at <22 weeks’ gestation, previous preterm delivery), and current pregnancy information (eg, use of ART). Data on the pregnancy course (eg, hospitalization during pregnancy, antenatal corticosteroid use for fetal maturation, threatened preterm labor, and type of onset of labor and delivery), pregnancy complications (eg, gestational hypertension, gestational diabetes, and other important pathologies), and neonatal issues were recorded. We decided not to include in the models the variables that are consequences of endometriosis.

In France, collecting data on race and ethnicity is strictly forbidden. Only information on the geographical origin of patients was collected.

### Main Outcomes, Sample Size, and Power Calculation

The primary outcome was a preterm birth between 22 weeks and 36 weeks and 6 days of gestation, regardless of the cause. The secondary outcomes were (1) spontaneous preterm birth between 22 weeks and 36 weeks and 6 days of gestation (or induced after premature rupture of membranes or chorioamnionitis), and (2) induced preterm birth between 22 weeks and 36 weeks and 6 days of gestation, which was defined as delivery after the induction of labor or a cesarean delivery before labor because of a maternal or a fetal indication. Other secondary outcomes were the occurrence of threatened preterm labor (defined as regular uterine contraction with cervical dilation before 34 weeks’ gestation, leading to hospitalization), premature rupture of membranes, SGA (defined as neonatal weight below the 10th percentile for gestational age^34^), gestational hypertension, preeclampsia (defined as gestational hypertension plus proteinuria ≥300 mg in a 24-hour urine collection^35^), placenta previa (as defined by D'Antonio and Bhide^36^), and postpartum hemorrhage (defined as blood loss ≥500 mL during the first 24 hours after delivery^37^).

The sample size was calculated to ascertain the number of patients required to demonstrate a doubling in the prevalence of preterm birth from 7% to 14% between the endometriosis and control groups. We needed to enroll 182 women in each phenotype group (SUP, OMA, and DE) to show a doubling of the risk for preterm birth compared with the control group (n = 1092; 2 control participants for each case with a preterm birth rate of 7%; β = 0.20; and α = .05).

### Statistical Analysis

We compared women in the endometriosis group with those in the control group by general characteristics; medical, surgical, and obstetric history; pregnancy course; and immediate neonatal issues and pregnancy complications. Women were compared for the primary outcome according to imaging and operative diagnoses of endometriosis.

We performed a multivariate analysis to determine whether there was an association between endometriosis and preterm birth (between 22 weeks and 36 weeks and 6 days of gestation) after adjustment for potential confounding factors (ie, risk factors reported in the literature as associated with preterm birth such as maternal age, body mass index before pregnancy, country of birth, parity, previous cesarean delivery, history of myomectomy, history of hysteroscopy, and preterm birth). We also conducted an analysis according to disease phenotype (SUP, OMA, and DE) to ascertain the association between endometriosis phenotype and the primary and/or secondary outcomes by comparing these 3 subgroups to the entire control group.

All of the statistical tests were 2-sided, and *P* < .05 was considered to be significant. All analyses were performed with SAS, version 9.4 (SAS Institute Inc) from October 7, 2020, to February 7, 2021.

## Results

### Study Participants

During the study period, we invited 1444 female patients in 7 maternity units to participate. After the exclusion of 93 women, 1351 women (mean [SD] age, 32.9 [5.0] years) who had a singleton delivery after 22 weeks’ gestation were retained for the analysis (Figure). No participants died during the study. For all participants, the deliveries occurred at the initially planned maternity unit.

**Figure.**
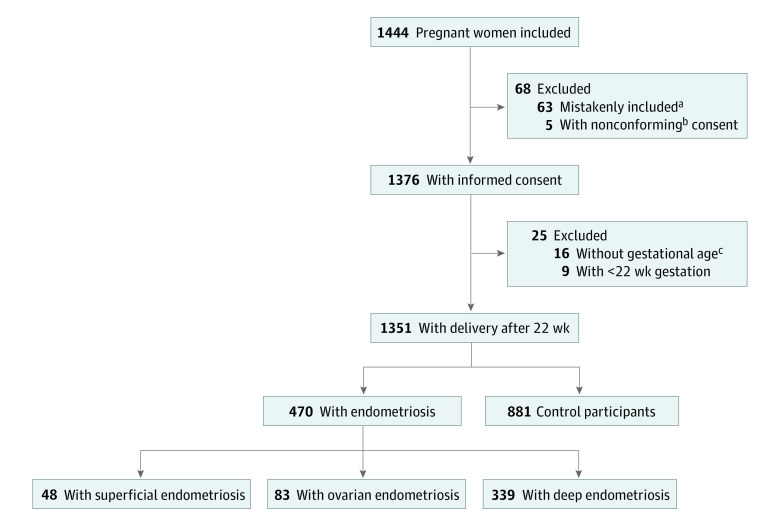
Participant Flowchart ^a^Of the 63 participants who were mistakenly included in the study, 22 did not have proof of a history of endometriosis (a surgical or imaging report), 29 did not meet the inclusion criteria, and 12 were from the control group who answered yes to 1 of the 3 anamnestic questions to suggest potential endometriosis. ^b^Of the 5 participants with nonconforming consent, 1 was from the endometriosis group and 3 were from the control group. ^c^Of the 16 participants for whom gestational age data were not available, 4 were from the endometriosis group and 12 were from the control group.

A total of 470 women were assigned to the endometriosis group (48 had SUP [10.2%], 83 had OMA [17.7%], and 339 had DE [72.1%]), and 881 women were assigned to the control group (Figure). Of the 339 women with DE, the anatomical distribution of the lesions was as follows: 306 uterosacral, 97 vaginal, 34 bladder, 146 bowel, and 26 ureteral (eTable 1 in the Supplement). Of the 470 women in the endometriosis group, 171 (36.4%) had an endometriosis diagnosis with in situ lesions according to imaging only, and 299 (63.6%) had an endometriosis diagnosis after surgical removal of histologically confirmed endometriotic lesions with possible residual endometriosis during pregnancy.

As shown in Table 1, the percentages of women who were 35 years or older, had a body mass index less than 18.5, and were native to Europe were higher in the endometriosis group than in the control group. The women with endometriosis were more likely than those without to have a history of myomectomy and operative hysteroscopy. They were also more often nulliparous with fewer cesarean deliveries, whereas the multiparous women had fewer preterm births between 22 and 36 weeks’ gestation. For current pregnancies, the women in the endometriosis group vs the control group were more likely to have had a longer median conception delay and greater requirement for ART to conceive. Issues that emerged during the pregnancies are presented in Table 2, with an increased rate of threatened preterm labor and cesarean deliveries observed (Table 2).

**Table 1.** Baseline Characteristics and Obstetric History of Participants

Characteristic	No./total No. (%)		*P* value^a^
	Endometriosis group	Control group	
All participants	470	881	NA
Age, y			
<30	90/470 (19.2)	213/881 (24.2)	.03
30-34	200/470 (42.5)	384/881 (43.6)	
≥35	180/470 (38.3)	284/881 (32.2)	
BMI			
<18.5	42/469 (9.0)	61/873 (7.0)	.02
18.5-24	315/469 (67.2)	567/873 (64.9)	
25-29	86/469 (18.3)	155/873 (17.8)	
≥30	26/469 (5.5)	90/873 (10.3)	
Ever use of tobacco	98/467 (21.0)	174/869 (20.0)	.68
Country of birth			
Europe	298/396 (75.2)	556/826 (67.3)	.002
North Africa	33/396 (8.3)	112/826 (13.6)	
Sub-Saharan Africa	18/396 (4.6)	70/826 (8.5)	
Other^b^	47/396 (11.9)	88/826 (10.6)	
Educational level			
No schooling; primary, middle, or vocational school attendance/completion	10/356 (2.8)	27/687 (3.9)	.61
High school diploma	36/356 (10.1)	64/687 (9.3)	
University/college degree (eg, IUT, DUT, higher education)	310/356 (87.1)	596/687 (86.8)	
Professional activity during pregnancy			
Working	351/400 (87.7)	678/778 (87.2)	.94
Not working	37/400 (9.3)	74/778 (9.5)	
Unemployed	12/400 (3.0)	26/778 (3.3)	
Medical-surgical history			
Preexisting			
Diabetes before pregnancy	4/469 (0.8)	18/880 (2.0)	.10
Hypertension before pregnancy	13/469 (2.8)	18/880 (2.0)	.40
History			
Myomectomy	14/467 (3.0)	11/879 (1.2)	.02
Dilatation and curettage	66/466 (14.2)	108/880 (12.3)	.33
Operative hysteroscopy	71/466 (15.2)	34/877 (3.9)	<.001
Obstetric history			
Gravidity, median (IQR), No.	1 (0-1)	1 (0-2)	<.001
0	232/469 (49.5)	268/880 (30.5)	<.001
1	142/469 (30.3)	252/880 (28.6)	
2	60/469 (12.8)	182/880 (20.7)	
≥3	35/469 (7.4)	178/880 (20.2)	
Parity and previous cesarean delivery			
Nulliparous	340/469 (72.5)	403/880 (45.8)	<.001
Multiparous			
Without previous cesarean delivery	82/469 (17.5)	329/880 (37.4)	
With previous cesarean delivery	47/469 (10.0)	148/880 (16.8)	
If multiparous, pregnancy history (at least 1), weeks’ gestation	129	477	
Pregnancy loss <22	54/129 (41.9)	241/477 (50.5)	.08
22-31	6/129 (4.6)	35/476 (7.3)	.28
32-36	12/129 (9.3)	53/476 (11.1)	.55
≥37	114/129 (88.4)	423/476 (88.9)	.87
Intrauterine fetal death at ≥22 weeks’ gestation	2/237 (0.8)	19/612 (3.1)	.06
Current pregnancy information			
Conception delay, median (IQR), mo	12 (3-36)	2 (1-6)	<.001
ART use	192/468 (41.0)	69/877 (7.9)	<.001

Abbreviations: ART, assisted reproductive technology; BMI, body mass index (calculated as weight in kilograms divided by height in meters squared); DUT, diplôme universitaire de technologie (French diploma after completion of 2-year college-level program); IUT, institute universitaire de technologie (French diploma after completion of 2-year college-level program); NA, not applicable.

^a^ *P* values were calculated using χ^2^ or Mann-Whitney test.

^b^ Other includes Asia, French Guiana, Guadeloupe, Martinique, Reunion Island, and missing data for the country or geographic region of birth.

**Table 2.** Pregnancy Course, Delivery, Immediate Postpartum Complication, and Neonatal Issue Among Participants

Variable	No./total No. (%)		*P* value^a^
	Endometriosis group	Control group	
All participants	470	881	NA
Pregnancy course			
Hospitalization during pregnancy	84/465 (18.1)	128/872 (14.7)	.11
Antenatal corticosteroid use for fetal maturation	38/464 (8.2)	50/872 (5.7)	.08
Threatened preterm labor	38/465 (8.2)	43/872 (4.9)	.02
PROM	14/465 (3.0)	24/873 (2.7)	.78
Preeclampsia or gestational hypertension	28/465 (6.0)	38/872 (4.4)	.18
Placental abruption	3/465 (0.6)	3/872 (0.3)	.42
Placenta previa	7/465 (1.5)	11/872 (1.3)	.71
Gestational diabetes	80/465 (17.2)	114/872 (13.1)	.04
Delivery and immediate postpartum complications			
Type of onset of labor			
Spontaneous	289/462 (62.5)	589/877 (67.2)	.06
Induction	125/462 (27.1)	187/877 (21.3)	
Cesarean delivery before labor	48/462 (10.4)	101/877 (11.5)	
Type of delivery			
Spontaneous	257/465 (55.3)	564/876 (64.4)	.003
Instrumental	81/465 (17.4)	109/876 (12.4)	
Cesarean delivery after labor	127/465 (27.3)	203/876 (23.2)	
Gestational age, median (IQR), weeks’ gestation	39 (38-40)	39 (38-40)	.79
<32	10/470 (2.1)	21/881 (2.4)	.42
32-36	24/470 (5.1)	32/881 (3.6)	
≥37	436/470 (92.8)	828/881 (94.0)	
Preterm delivery between 22-36 weeks’ gestation	34/470 (7.2)	53/881 (6.0)	.38
If yes, type			
Spontaneous	19/32 (59.4)	30/53 (56.6)	.80
Induced	13/32 (40.6)	23/53 (43.4)	
Perioperative complication of cesarean delivery	7/124 (5.6)	6/198 (3.0)	.25
PPH	40/462 (8.7)	51/875 (5.8)	.05
If yes, cause			
Uterine atony	16/40 (40.0)	30/51 (58.8)	.07
Placental retention	8/40 (20.0)	8/51 (15.7)	.59
Cervicovaginal injury	6/40 (15.0)	9/51 (17.6)	.73
Other^b^	10/40 (25.0)	9/50 (18.0)	.42
Peripartum			
Hysterectomy	1/40 (2.5)	0/51 (0)	.44
Transfusion	10/40 (25.0)	5/51 (9.8)	.05
Postpartum endometritis	2/457 (0.4)	5/872 (0.6)	.99
Infection of the operative site	2/457 (0.4)	1/872 (0.1)	.27
Neonatal issue			
Live birth	462/465 (99.4)	875/878 (99.7)	.42
Male sex	243/464 (52.4)	452/876 (51.6)	.79
Female sex	221/464 (47.6)	424/876 (48.4)	
Birth weight, median (IQR), g	3190 (2920-3510)	3310 (3020-3620)	<.001
SGA^c^	72/462 (15.6)	77/875 (8.8)	<.001
Apgar at 5 min <7	6/457 (1.3)	18/871 (2.1)	.33
Arterial pH<7.10	28/447 (6.3)	49/846 (5.8)	.73
Delivery room resuscitation	35/458 (7.6)	52/872 (6.0)	.24
Immediate transfer to neonatal unit	34/459 (7.4)	47/874 (5.4)	.14

Abbreviations: NA, not applicable; PPH, postpartum hemorrhage; PROM, premature rupture of membranes; SGA, small for gestational age.

^a^ *P* values were calculated using χ^2^ or Mann-Whitney test.

^b^ Other causes included the following: unavailable data (n = 6), uterine scar injury (n = 4), perineal injury (n = 5), uterine artery injury (n = 2), maternal severe thrombopenia with coagulopathy (n = 1), and postcesarean immediate hemoperitoneum (n = 1).

^c^ According to EPOPé (Obstetrical Perinatal and Pediatric Epidemiology Research Team) intrauterine growth curves; defined as neonatal weight below the 10th percentile for gestational age.^34^

### Primary Outcome

No difference was observed for the median gestational age and the rate of preterm delivery before 37 weeks’ gestation between the endometriosis group and the control group (34 of 470 [7.2%] vs 53 of 881 [6.0%]; *P* = .38) (Table 2). In addition, no difference was observed regarding the type of preterm birth (spontaneous or induced).

### Secondary Outcomes

The rates of gestational diabetes (80 of 465 [17.2%] vs 114 of 872 [13.1%]; *P* = .04) and SGA (72 of 462 [15.6%] vs 77 of 875 [8.8%]; *P* < .001) were significantly higher in the endometriosis group compared with the control group (Table 2). No differences were observed for the other secondary outcomes. No differences were observed regarding immediate neonatal issues except for the median (IQR) birth weight (3190 [2920-3510] g vs 3310 [3020-3620] g; *P* < .001), which was significantly lower for the endometriosis group than the control group (Table 2).

After adjusting for potential confounding factors, we observed that endometriosis was not associated with preterm birth (adjusted odds ratio [aOR], 1.07; 95% CI, 0.64-1.77), and no association was found for the secondary outcomes except for threatened preterm labor (aOR, 1.81; 95% CI, 1.08-3.04) (Table 3). The primary and secondary outcomes were also compared with the control group according to endometriosis phenotype, and they did not differ (SUP: 6.2% [3 of 48]; OMA: 7.2% [6 of 83]; and DE: 7.4% [25 of 339]; *P* = .84) except for SGA (aOR, 1.45; 95% CI, 0.99-2.10), which was increased in cases of OMA or DE (eTable 2 in the Supplement). In addition, no difference was observed for the primary outcome between patients with an endometriosis diagnosis by imaging and patients with a complete lesion removal or pathological documentation (9 of 171 [5.3%] vs 25 of 299 [8.4%]; *P* = .21).

**Table 3.** Association Between Endometriosis and Primary and Secondary Outcomes

Outcome	OR (95% CI)	
	Crude	Adjusted^a^
Preterm birth at <37 weeks’ gestation	1.22 (0.78-1.90)	1.07 (0.64-1.77)
Spontaneous delivery	1.20 (0.67-2.16)	0.89 (0.45-1.74)
Induced delivery	1.07 (0.53-2.12)	1.23 (0.58-2.61)
Threatened preterm labor	1.72 (1.09-2.69)	1.81 (1.08-3.04)
PROM at <37 weeks’ gestation^b^	1.10 (0.56-2.14)	1.02 (0.50-2.11)
SGA^b^	1.91 (1.36-2.70)	1.45 (0.99-2.10)
Preeclampsia^c^	1.30 (0.60-2.82)	0.86 (0.35-2.11)
Placenta previa^d^	1.20 (0.46-3.11)	1.28 (0.43-3.76)
PPH^e^	1.53 (1.00-2.35)	1.41 (0.88-2.23)

Abbreviations: OR, odds ratio; PPH, postpartum hemorrhage; PROM, premature rupture of membranes; SGA, small for gestational age (defined as neonatal weight below the 10th percentile for gestational age^34^).

^a^ OR was adjusted for maternal age, body mass index before pregnancy, country of birth, parity and previous cesarean delivery, history of myomectomy, history of hysteroscopy, and history of preterm birth (22-36 weeks’ gestation).

^b^ According to EPOPé (Obstetrical Perinatal and Pediatric Epidemiology Research Team) intrauterine growth curves.^34^

^c^ Attributed to a convergence problem; defined as gestational hypertension plus proteinuria ≥300 mg in a 24-hour urine collection.^35^

^d^ Attributed to a convergence problem; defined by D'Antonio and Bhide.^36^

^e^ Attributed to a convergence problem; defined as blood loss ≥500 mL during the first 24 hours after delivery.^37^

## Discussion

In this large, prospective cohort study, the rate of preterm birth before 37 weeks’ gestation was not different between women with vs women without endometriosis. No difference according to the disease phenotype was found.

These findings are not in accordance with results of studies on the increased risk for preterm delivery in cases of endometriosis, including results of the 3 main meta-analyses of the obstetric outcomes in patients with endometriosis.^15,16,17^ These studies found an association between endometriosis and adverse pregnancy outcomes, especially preterm birth before 37 weeks’ gestation (OR, 1.63 [95% CI, 1.32-2.01]^15^; OR, 1.70 [95% CI, 1.40-2.06]^16^; and OR, 1.38 [95% CI, 1.01-1.89]^17^). However, the results were not consistent among the included studies for the association between endometriosis and preterm birth (OR, 1.30 [95% CI, 1.22-1.40]^20^; OR, 1.34 [95% CI, 1.15-1.56]^19^; OR, 1.15 [95% CI, 0.91-1.45]^38^; and OR, 1.14 [95% CI, 0.87-1.51]^39^). These meta-analyses were potentially biased, however, because of small-study effect sizes,^40^ and there was a potential publication bias given that studies showing no difference were less likely to be published.^41^ Furthermore, the diagnosis of endometriosis and the selection of the comparison group were not uniform across the studies. A retrospective cohort study has addressed the question of the implication of the disease phenotype for perinatal outcomes.^42^ The investigators found fewer preterm births only in cases of isolated OMA, and there was a higher incidence of placenta previa associated with rectovaginal DE lesions.^42^

The increased use of ART and conception delay in the endometriosis group were not included in the multivariate analysis of the present study to avoid overadjustment given that both are inherently associated with endometriosis and are known to be associated with preterm birth.^43^ Despite this association, in the present study, endometriosis was not associated with preterm birth. Endometriosis is a heterogeneous disease comprising 3 distinct phenotypes and is associated with adenomyosis in 30% of cases.^44^ Such heterogeneity could have implications for the perinatal outcome of endometriosis.

This study did not find that women with endometriosis had an increased risk for preterm birth. However, because we observed a higher rate of threatened preterm labor in the endometriosis group, we cannot exclude an association between endometriosis, uterine contractions, and cervical changes.^45^ Practitioners also often mistakenly designate pregnancies of women with endometriosis as high risk because of (1) available retrospective data on adverse pregnancy outcomes, (2) known rare but potentially catastrophic acute surgical complications during pregnancy that were related to DE (hemoperitoneum, uroperitoneum, intestinal perforation, and uterine rupture),^21^ (3) an extensive surgical history, or (4) a lengthy infertility. This anxiety-provoking setting could explain the association between endometriosis and hospitalization for threatened preterm labor (despite the absence of an association with increased risk of preterm birth). The higher rate of threatened preterm labor could be attributed to an indication bias associated with decisions on overindicated measures to manage threatened preterm labor, such as hospitalization during pregnancy. Although hospitalization and tocolysis during pregnancy have been associated with prolonged pregnancy by a few days (48 hours to 7 days), no studies have demonstrated an association with a reduced rate of preterm birth.^46^ We were not surprised to observe a higher rate of induction of labor, instrumental delivery, and cesarean delivery in the endometriosis group, possibly because of an overestimation of high-risk pregnancies in cases of endometriosis.

These results may provide a degree of reassurance to patients and encourage practitioners to monitor such pregnancies according to normal pregnancy protocols to prevent preterm birth, keeping in mind the exceptional gestational acute surgical complications. Further studies are warranted to identify other adverse perinatal outcomes or specific but rare complications.

### Strengths and Limitations

This study has some strengths. Its main strength relates to the prospective enrollment of participants. The attending practitioners prospectively collected the data, and the specific characterization of the history of endometriosis was based on imaging, surgical, and pathological reports, which allowed the nature of the disease to be characterized as precisely as possible. Although the women in the control group did not have an imaging workup before pregnancy, we avoided the enrollment of women who could have had unknown endometriosis by posing questions before inclusion that are known to be specific for endometriosis.^1^ Women who were referred to the participating maternity units were not included to avoid selection bias given that these patients were at high risk for preterm birth. In addition, this study was designed to provide sufficient statistical power in an unselected population.

This study also has several limitations. First, as is the case with all observational studies, this study had uncontrolled confounders despite the multivariate analysis. It is also possible that the control group did not represent a low-risk pregnancy group from the general population. However, the frequency of preterm birth in the control group was comparable to the frequency observed in the general population in France.^47^ Second, the number of included women was low because of recruitment difficulties (470 women in the endometriosis group instead of the intended 546, and 881 in the control group instead of the intended 1092). The recruitment was not stratified according to the endometriosis phenotype, thereby resulting in the observed imbalance between the 3 phenotype groups. However, with 339 women included, the study ensured adequate power for the DE phenotype but not for the SUP and OMA phenotypes. In addition, we assumed that we did not reach the exact expected power to fully assess the implication of the disease phenotype, although the available data did not reveal any pattern except for SGA, which was increased in the OMA and DE phenotypes, thus calling for further analysis.

Third, imaging diagnoses of endometriosis raise the question of diagnostic confirmation of endometriosis. It is now well documented that specific imaging criteria are sufficiently accurate for a nonsurgical diagnosis of OMA and DE.^1,48^ However, the endometriosis group included women with a diagnosis based on imaging as well as women who were surgically treated for endometriosis. This heterogeneity could be a bias given the presupposed benefit of removing ectopic lesions for a viable intrauterine pregnancy,^49^ which is different from an adverse pregnancy outcome. This point does not affect the interpretation of the results of this study because no difference in the rate of preterm birth was observed between the women with a diagnosis based on imaging and those who underwent a complete removal of their lesions. In addition, women with endometriosis more often have a history of gynecological issues (ie, myomectomy and operative hysteroscopy), which is why we included this parameter in the multivariate analysis.

Fourth, we did not provide 3 phenotype groups with isolated lesions (superficial ovarian or deep lesions) because they are often associated. However, endometriosis is a heterogeneous disease,^50^ and SUP is frequently associated with OMA or DE and cannot be seen on imaging. In addition, when associated with painful symptoms, OMA is associated with DE.^51^ The extent to which SUP plays a role in obstetric outcomes vs OMA or DE remains unknown.

## Conclusions

This multicenter, prospective cohort study found that endometriosis was not associated with preterm birth and that the disease phenotype did not appear to interfere with the result. Pregnant women with endometriosis should not be considered to have an exceptionally high risk for preterm birth; thus, monitoring their pregnancy beyond the normal protocols or changing management strategies may not be warranted. However, further studies are needed to examine the potential for other adverse perinatal outcomes or specific but rare complications.

## Notes

Supplement. **eTable 1.** Diagnosis Modalities and Endometriosis Phenotype **eTable 2.** Primary and Secondary Outcomes According to the Endometriosis Phenotype
