# Supplementary material for: Association Between Endometriosis Phenotype and Preterm Birth in France
Source: JAMA Netw Open. 2022 Feb 8;5(2):e2147788. doi: 10.1001/jamanetworkopen.2021.47788 (PMC8826171; doi:10.1001/jamanetworkopen.2021.47788)
Supplement: Supplement. — eTable 1. Diagnosis Modalities and Endometriosis Phenotype eTable 2. Primary and Secondary Outcomes According to the Endometriosis Phenotype [file jamanetwopen-e2147788-s001.pdf]

Supplementary Online Content

Marcellin L, Goffinet F, Azria E, et al. Association between endometriosis phenotype and preterm birth in France. *JAMA Netw Open*. 2022;5(2):e2147788. doi:10.1001/jamanetworkopen.2021.47788

eTable 1. Diagnosis Modalities and the Endometriosis Phenotype

eTable 2. Primary and Secondary Outcomes According to the Endometriosis Phenotype

This supplementary material has been provided by the authors to give readers additional information about their work.

© 2022 Marcellin L et al. *JAMA Network Open.*

eTable 1. Diagnosis Modalities and the Endometriosis Phenotype

	**Endometriosis**					
	**SUP**		**OMA**		**DE**	
	(n = 48)		(n = 83)		(n = 339)	
	n/N	%	n/N	%	n/N	%
Imaging workup	25/47	53.2	66/83	79.5	314/339	92.6
Surgery for endometriosis	48/48	100	49/83	59.0	204/339	60.2
Associated adenomyosis						
none	34/43	79.1	62/73	84.9	145/288	50.3
Diffuse	6/43	13.9	2/73	2.8	54/288	18.8
Focal	3/43	7.0	9/73	12.3	77/288	26.7
Diffuse and focal	0		0		12/288	4.2
Anatomical distribution of deep lesions						
Uterosacral ligament					306/333	91.9
Vagina					97/327	29.7
Bladder					34/326	10.4
Bowel					146/332	44.0
Ureter					26/327	7.9

SUP : superficial endometriosis, OMA endometrioma, DE deep endometriosis

© 2022 Marcellin L et al. *JAMA Network Open.*

eTable 2. Primary and Secondary Outcomes According to the Endometriosis Phenotype

	Endometriosis								
	**Superficial**		**Ovarian**		**Deep**		**Controls**		
	(n = 48)		(n = 83)		(n = 339)		(n = 881)		
	n/N	%	n/N	%	n/N	%	n/N	%	p
Preterm birth < 37 WG	3/48	6.2	6/83	7.2	25/339	7.4	53/881	6.0	.84
Spontaneous preterm birth	1/47	2.1	4/83	4.8	14/338	4.1	30/881	3.4	.79
Induced preterm delivery	1/47	2.1	2/83	2.4	10/338	2.9	23/881	2.6	.97
Threatened preterm labor	5/47	10.6	6/82	7.3	27/336	8.0	43/872	4.9	.07
Preterm premature rupture of membranes < 37 WG	0/47	0.0	2/82	2.4	12/336	3.6	24/873	2.7	.68
Small for gestational age*	5/47	10.6	17/82	20.7	50/333	15.0	77/875	8.8	< .001
Preeclampsia	2/47	4.3	2/82	2.4	7/336	2.1	16/872	1.8	.46
Placenta previa	0/47	0.0	1/82	1.2	6/336	1.8	11/872	1.3	.89
Postpartum hemorrhage	5/47	10.6	9/82	11.0	26/333	7.8	51/875	5.8	.16

P: Chi^2 or Fisher's exact test between the four groups
* according to EPOPE Curves

© 2022 Marcellin L et al. *JAMA Network Open.*
